# Orientation Ordering and Chiral Superstructures in Fullerene Monolayer on Cd (0001)

**DOI:** 10.3390/nano10071305

**Published:** 2020-07-03

**Authors:** Yuzhi Shang, Zilong Wang, Daxiao Yang, Yaru Wang, Chaoke Ma, Minlong Tao, Kai Sun, Jiyong Yang, Junzhong Wang

**Affiliations:** School of Physical Science and Technology, Southwest University, Chongqing 400715, China; shangyuzhi@swu.edu.cn (Y.S.); fragment@email.swu.edu.cn (Z.W.); yangdx@email.swu.edu.cn (D.Y.); wyr19951221@email.swu.edu.cn (Y.W.); children999@email.swu.edu.cn (C.M.); taotaole@swu.edu.cn (M.T.); skqtt@swu.edu.cn (K.S.); jyyang@swu.edu.cn (J.Y.)

**Keywords:** STM, C_60_, chiral motifs, orientational ordering

## Abstract

The structure of C_60_ thin films grown on Cd (0001) surface has been investigated from submonolayer to second monolayer regimes with a low-temperature scanning tunneling microscopy (STM). There are different C_60_ domains with various misorientation angles relative to the lattice directions of Cd (0001). In the (2√3 × 2√3) R30° domain, orientational disorder of the individual C_60_ molecules with either pentagon, hexagon, or 6:6 bond facing up has been observed. However, orientation ordering appeared in the R26° domain such that all the C_60_ molecules adopt the same orientation with the 6:6 bond facing up. In particular, complex chiral motifs composed of seven C_60_ molecules with clockwise or anticlockwise handedness have been observed in the R4° and R8° domains, respectively. Scanning tunneling spectroscopy (STS) measurements reveal a reduced HOMO–LOMO gap of 2.1 eV for the C_60_ molecules adsorbed on Cd (0001) due to the substrate screening and charge transfer from Cd to C_60_ molecules.

## 1. Introduction

As a prototypical fullerene molecule, C_60_ can be used as the building blocks for carbon-based nanomaterials. A variety of C_60_ monolayers grown on solid surfaces is critical for understanding and controlling the interface properties of fullerene-derived electronic and photovoltaic devices [1,2,3]. In the past decades, there have been a large number of investigations of C_60_ monolayer structures grown on a wide range of metallic or semiconducting substrates such as Ag [4,5,6,7,8,9,10,11], Au [12,13,14,15,16,17,18,19,20,21,22,23], Cu [24,25,26,27,28], graphene [29,30,31,32,33], Si [34,35,36], Ge [37,38,39], or NaCl [40]. It was found that almost all monolayers of C_60_ adopt a close-packed structure regardless whether the substrate is isotropic or anisotropic, metallic or nonmetallic.

The C_60_ domains can take different orientations with respect to the directions of substrate lattices. On noble metal surfaces such as Au(111) or Ag(111), STM studies have demonstrated that the C_60_ monolayer domains exhibit a variety of lattice orientations such as the “in phase” (7 × 7) R0° [21], (2√3 × 2√3) R30° [4,8,9,10,18,19,20,21,22], and (√589 × √589) R14.5° [12,14,21] phases, which contain 4, 1, and 49 molecules per unit cell, respectively. In particular, “bright” and “dim” molecules have been found in the (2√3 × 2√3) R30° commensurate domains. It was proposed that the dim molecules are located on single atom vacancies, while the bright molecules remain on top position of the unreconstructed Au (111) surface. Moreover, a uniform R30° domain was also observed for all C_60_ molecules with the same contrast [15,21].

In addition to the different close-packing directions, the orientations of individual molecules within a single domain can also be different. The individual C_60_ molecules within a single domain can display multiple orientations such that the orientational ordering can be observed at 78 K [35]. For example, a complex orientational ordering was observed for molecules inside the “in-phase” (R0°) domain after room-temperature deposition, where a 7-molecule cluster is composed of a central molecule and six tilted surrounding molecules [18]. In the R14.5° domain, there are 49 molecules with a number of different orientations, on average, every time the seventh C_60_ appeared dim, giving rise to a quasiperiodic 7 × 7 superstructure at 5.7 K [12]. In particular, a chiral superstructure made of 7-molecule pinwheels was identified in C_60_ multilayer film on NaCl at 77 K [40] and in K_x_C_60_ monolayer on Au (111) at 7 K [41]. The proposed mechanism for orientational ordering was attributed to the maximized overlapping of neighboring molecular orbitals driven by the superexchange interaction. The latter refers to virtual hopping of an electron from the HOMO of one $C_{60}^{4-}$ to the LUMO of its nearest neighbor to gain an energy proportional to the hopping amplitude and the inverse of HOMO–LUMO gap.

In this paper, we chose the divalent metal Cd (0001) thin films as the substrate. The hexagonal close-packed metal Cd is usually used as an electrode material due to the smaller electronegativity compared with precious metals [42,43,44]. Thus, it would be fundamentally important to study the interfaces structure of C_60_ monolayer on Cd (0001) because the C_60_ molecules need to be contacted with metallic electrode in the electronic devices. It is found that the C_60_ domains exhibit a variety of orientations with respect to the lattice directions of Cd (0001). In the (2√3 × 2√3) R30° domain, individual C_60_ molecules reveal orientational disorder with the pentagon, hexagon, or 6:6 bond facing up. In the R26° domain, orientation ordering takes place such that all the C_60_ molecules adopt the same orientation with the 6:6 bond facing up. More interestingly, the complex chiral motifs composed of seven C_60_ molecules with clockwise or anticlockwise handedness have been observed in the R4° and R8° domains. STS measurements shows that the C_60_ molecules have a reduced HOMO–LOMO gap of 2.1 eV, indicating a significant charge transfer from Cd to C_60_ and strong substrate screening effect.

## 2. Experiment

The experiments were conducted in an ultra-high vacuum, low-temperature scanning tunneling microscopy (Unisoku USM1500) with a base pressure 1.2 × 10^−10^ mbar. The Cd (0001) thin films were grown on the Si (111)-7 × 7 surface at room temperature. The Si (111) substrate was continuously degassed at ~870 K for 8 h with subsequent flashing to 1400 K for several seconds. Cd atoms with a purity of 99.998% were thermally evaporated onto the Si (111) substrate from a quartz crucible by controlling the current. Deposition of 15 monolayer (ML) Cd at room temperature results in flat and smooth Cd (0001) films, which exhibit a perfect transparency due to the strong anisotropic electron motion with large lateral effective mass [45]. C_60_ were evaporated from a homemade tantalum boat at a rate of 0.4 ML/min with the temperature of 770 K. After the deposition, the sample was transferred into the LT-STM chamber. All the STM images were obtained in constant-current mode at 78 K. All differential conductance *dI/dV* spectra were acquired using a standard lock-in amplifier with a bias modulation signal of 10 mV at 1999 Hz under open-loop condition.

## 3. Results and Discussion

**Figure 1:**
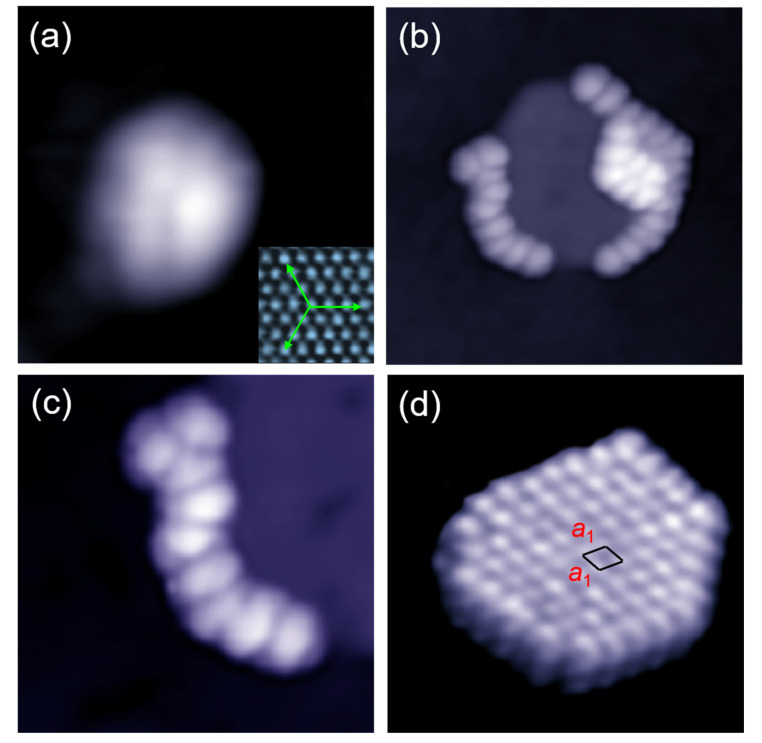
Initial stage of the self-assembly of C_60_ molecules on Cd (0001). (**a**) STM image of an isolated C_60_ molecule on Cd(0001), 4.5 nm × 4.5 nm, 1.0 V, 28 pA. Inset is the hexagonal lattices of the Cd(0001) thin film grown on the Si(111)-7 × 7 surface, 2 nm × 2 nm, -0.05 V, 35 pA. (**b**) Step decoration of C_60_ molecules to a round Cd island, 20 nm × 20 nm, 1.7 V, 25 pA. (**c**) Close-up view of the C_60_ chain attaching to the edge of Cd island, 10 nm × 10 nm, 0.5 V, 25 pA. (**d**) A monolayer island of C_60_ formed on the Cd substrate, 15.7 nm × 15.7 nm, 2.2 V, 28 pA.

We firstly studied the initial adsorption stage of C_60_ molecules on Cd (0001). Figure 1a shows the STM image of an isolated C_60_ molecule, which has a faint nodal plane separating the round protrusion into two lobes. The insert in Figure 1a shows the atomic-resolution image of the hexagonal lattices of Cd (0001) thin films grown on Si(111)–7 × 7 The measured lattice constant of Cd (0001) films is 3.0 ± 0.05 Å, very close to the bulk value (2.973 Å) in Cd crystals [44]. Figure 1b shows the nucleation of C_60_ molecules around a 2D Cd island. It was found that the C_60_ molecules aggregated at the step edge of the Cd island, due to the increased interaction with substrate at these positions. From the zoomed-in image in Figure 1c, anisotropy can be found from the individual C_60_ molecules attaching to the Cd island: most molecules show an anisotropic shape due to the tip convolution effect in the fast-scanning direction, while keep the original length (~1 nm) parallel to the step edge. In fact, such a type of step decoration was previously observed in the C_60_ nanostructures grown on metal surfaces. The C_60_ molecules initially adsorbed at intersections of multiple steps and edges of monatomic steps on narrow terraces and periodic arrays of short chains formed as the coverage is increased [46]. When C_60_ molecules attach on Ag (111) island, a complete, single-strand C_60_ “necklace” circling the island formed. The decoration, in turn, made the equilibrium island shape round [47]. Shown in Figure 1d is a monolayer island of C_60_ with a close-packed hexagonal structure. There is a misorientation angle of 20° between the lattice directions of C_60_ island and Cd (0001) surface. The error limit in the measurement of orientation angle is ±2° in our experiments. The measured lattice constant is $a_{1}=10.2\pm 0.1\;Å\;$, larger than the lattice constant (10.02 Å) of the (111) plane in *fcc* C_60_ crystals [48]. The apparent height of C_60_ molecules in this island is measured to be 7.0 ± 0.1 Å, at the bias of 2.2 V.

**Figure 2:**
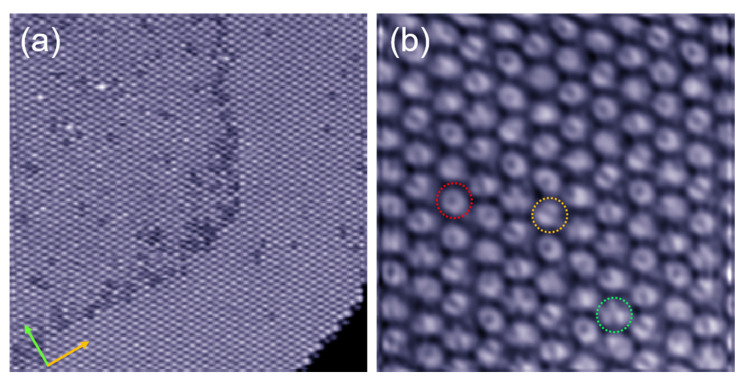
STM images of the (2√3 × 2√3) R30° domain of C_60_ island on Cd (0001). (**a**) Uniform domain of the R30° with all C_60_ molecules in the same contrast except a few dim molecules appeared randomly, 50 nm × 50 nm, 2.2 V, 29 pA. The green and yellow arrows mark the directions of base vector and R30° of the Cd (0001), respectively. (**b**) Close-up view of the uniform domain without any dim molecules, 10 nm × 10 nm, 1.0 V, 18 pA. The red, green, and yellow circles mark the C_60_ molecules with a pentagon, hexagon, and 6:6 bond facing up, respectively.

When the coverage increased to one monolayer, C_60_ molecules form a close-packed (2√3 × 2√3) R30° domain as shown in Figure 2a. The apparent height of C_60_ molecules in this domain is measured to be 9.0 ± 0.1 Å, at the bias of 2.2 V. The measured intermolecular spacing is increased to 10.4 ± 0.1 Å, much larger than the preferred spacing of in C_60_ crystals [48]. It implies that all the C_60_ molecules in the (2√3 × 2√3) R30° domain suffer a tensile strain as large as 3.8%, which would make the R30° domain less stable. Moreover, we noticed that all the C_60_ molecules present a uniform height except a few dim molecules. The height difference between the dim and normal C_60_ molecules is ~0.6 Å. In particular, some dim molecules aggregate into a wire-like region, indicating the attractive force among the dim molecules. Figure 2b is the high-resolution STM image of the uniform R30° domain, where C_60_ molecules with different molecular orientations can be identified. The individual molecules marked by red, green, and yellow circles exhibit a round protrusion with a small hole (like a doughnut), three lobes like a clover, and two parallel lobes corresponding to the C_60_ molecules with a pentagon, hexagon, and 6:6 bond facing up, respectively [13,40]. We noticed that such types of orientational disorder also appeared in the R30° domains of C_60_ grown on Au (111) [13,18,21,22,23] or Ge (111) surfaces [39].

**Figure 3:**
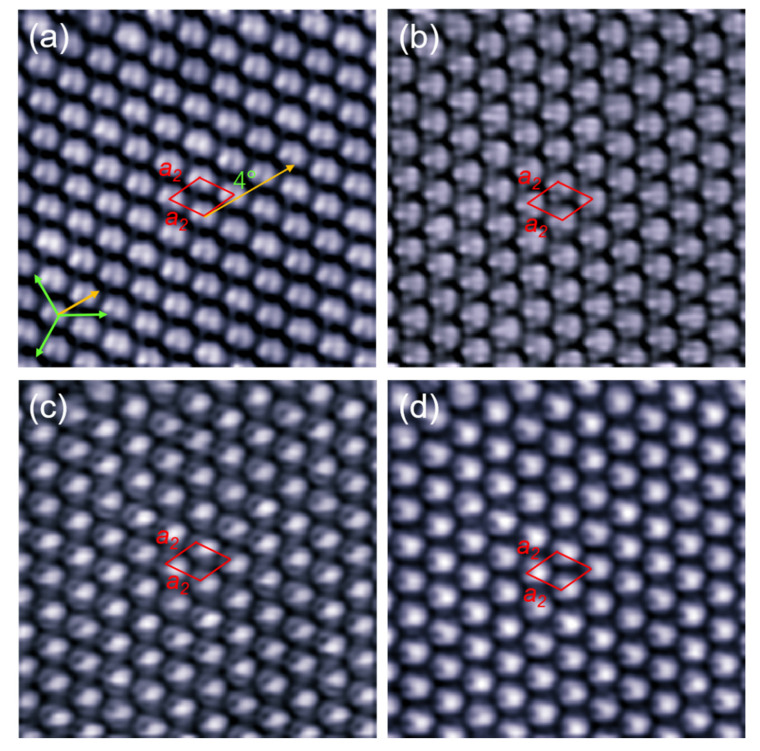
Bias-dependent STM images (10 nm × 10 nm) of the C_60_ monolayer in the R26° domain. The bias voltages in (**a**–**d**) are 1.4, 0.6, −1.4, and −2.0 V, respectively. All the tunneling currents are 30 pA. All the C_60_ molecules are arranged in the same orientation. The individual C_60_ molecules reveal sub-molecular contrast such as the two parallel lobes in (a), a small hole and a bright oval in (c), and a bright ball with small opening in (d).

When the lattice direction of C_60_ monolayer deviates from the Cd (0001) lattice for 26° angle, all C_60_ molecules adopt the same orientation, forming a homogeneous orientation domain. It is observed that from the empty-state STM image at 1.4 V (Figure 3a), all the C_60_ molecules present a two-lobe structure oriented at the same direction, similar to the motifs marked by yellow circle in Figure 2b. It means that all the molecules in R26° domain adopt the 6:6 bond facing-up orientation, and are adsorbed at the equivalent sites of the Cd (0001). The apparent height of C_60_ molecules in this domain is measured to be 8.7 ± 0.1 Å at the bias of −2.8 V. The intermolecular spacing is 10 ± 0.2 Å, slightly smaller than that in the C_60_ island of Figure 1d. Although a similar phenomenon was observed previously in the close-packed C_60_ monolayer grown on Au (111) [21], the bias-dependent behaviors of the homogeneous domain has not been studied yet. Here, we have made investigations of the variation of C_60_ molecular contrast with the bias voltage from 1.4 to −2.0 V in the R26° domain. As shown in Figure 3b, when the bias is reduced to 0.6 V, the contrast of each molecule shows four small protrusions: three bright and one dim. In the filled state STM image at −1.4 V (Figure 3c), all the C_60_ molecules present a bright protrusion but with a small off-center hole, resembling the contrast of C_60_ molecule with a carbon atom at the top [35,49]. When the bias voltage is increased to −2.0 V, the off-center hole becomes smaller and the bright protrusion becomes larger (Figure 3d). It is well known that STM image yields a spatial map of the local density of states (DOS) for the adsorbates on metal surfaces. Thus, the bias-dependent STM images mentioned above can be attributed to the DOS variation of C_60_ molecules with energy. However, the possibility of orientation modification driven by the tip electric field cannot be safely excluded under the successive STM scanning. Previously, the tip-induced rotation was reported in the unstable C_60_ molecules adsorbed on graphene/Cu (111), where tip electron field or tunneling electron might play an important role for the reorientation [32].

**Figure 4:**
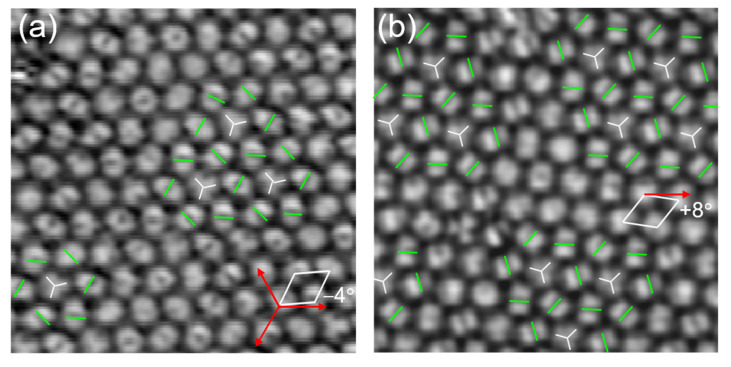
Chiral pinwheel patterns appeared in the R (−4°) and R (+8°) domains. (**a**) Seven C_60_ molecules with different directions constitute a chiral pinwheel motif with clockwise handedness in the R (−4°) domain, 10 nm × 10 nm, 2.0 V, 28 pA. There is a central molecule with three-lobe protrusion. (**b**) Chiral pinwheels with anticlockwise handedness in the R (+8°) domain, 10 nm × 10 nm, 2.2 V, 22 pA.

Beside the homogeneous orientation, we also found a local and complex orientational ordering in the C_60_ monolayer domain. As shown in Figure 4a, four chiral motifs composed of 7 C_60_ molecules appeared in the R (−4°) domain. The central C_60_ molecule presents three lobe protrusions, the other six tilted surrounding molecules appear as two-lobe motifs with a nodal plane aligned at different directions, constituting a pinwheel pattern with clockwise handedness. Figure 4b shows a C_60_ domain with a misorientation angle of +8° relative to the lattice directions of Cd (0001). There are eleven chiral pinwheels with anticlockwise handedness in this domain. Such types of 7-molecule clusters resemble the C_60_ clusters appeared in the R0° domain grown on Au (111) [18], but the chiral feature appeared in the present domain. To some extent, it is more like the chiral motifs appeared in the C_60_ multilayer on NaCl [40] or K_4+__δ_ C_60_ monolayer on Au (111) surface [41], which was attributed to overlap of neighboring molecular orbitals due to the superexchange interaction. The latter refers to virtual hopping of an electron from the HOMO of one $C_{60}^{4-}$ to the LUMO of its nearest neighbor to gain an energy proportional to the hopping amplitude and the inverse of HOMO–LUMO gap. Both the apparent heights of C_60_ molecules in these two domains are 9.5 ± 0.1 Å. Furthermore, we found the C_60_ molecules in above two domains reveal the same intermolecular distance as that in Figure 3, i.e., 10 ± 0.2 Å.

**Figure 5:**
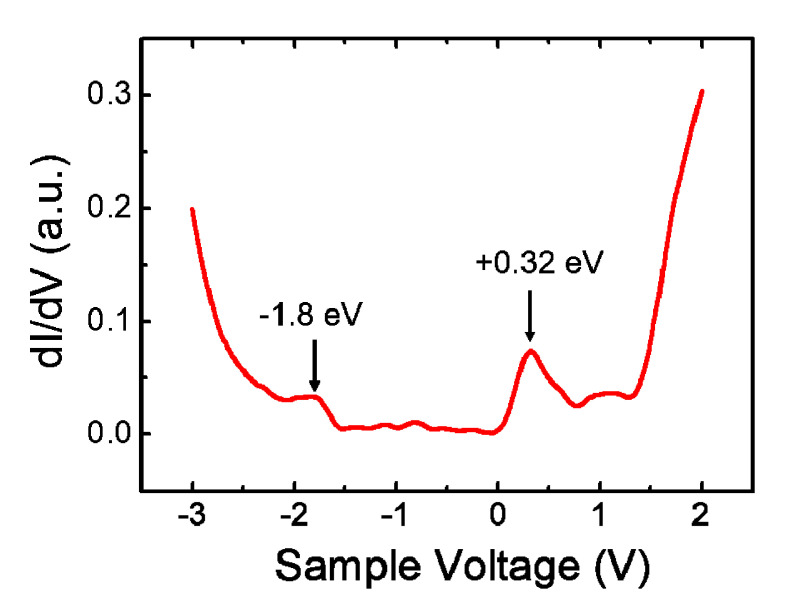
dI/dV spectra acquired on top of a C_60_ molecule in the (2√3 × 2√3) R30° domain. The HOMO–LUMO gap is 2.12 eV. Tunneling parameters were *U* = 0.9 V, *I* = 66 pA before taking the spectra.

Figure 5 shows a scanning tunneling spectra (STS) acquired on top of a C_60_ molecule at 77 K in the (2√3 × 2√3) R30° domain. The measured highest occupied molecular orbital (HOMO) is located at −1.8 eV, while the lowest unoccupied molecular orbital (LUMO) appears at 0.32 eV. Thus the HOMO–LUMO gap is 2.12 eV, which is much smaller than that (4.9 eV) of a free C_60_ molecule in gas state, but comparable to that (2.7 eV) of C_60_ on Au (111) and (2.3 eV) of C_60_ on Ag (100) [5,13]. Compared with the LUMOs of C_60_ on noble metal surfaces [0.84 eV for Au (111), 0.49 eV for Ag (100), and 0.43 eV for Cu (100)], the lower position of C_60_ LUMO (0.32 eV) on Cd (0001) reflect the stronger reactivity of Cd substrate [4,5,13]. Moreover, the effect of substrate screening may also reduce the HOMO–LUMO gap, through reducing the intramolecular Coulomb repulsion [50]. In addition to the peaks corresponding to HOMO and LUMO, there are also small peaks appeared in the gap, which result from the surface states or quantum well state in the Cd (0001) films [45].

**Figure 6:**
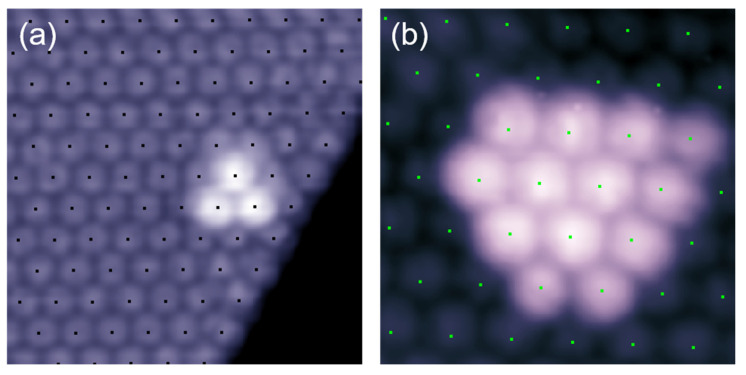
C_60_ clusters on top of the first layer. (**a**) A C_60_ trimer appeared in the second layer with each molecule located at the atop site, 2.6 V, 10 nm × 10 nm, 25 pA. (**b**) A large cluster with thirteen molecules in the second layer, 2.0 V, 6 nm × 6 nm, 30 pA. The individual molecules within clusters are essentially located at the atop position of the first layer.

We have also studied the structure of the second C_60_ layer. Shown in Figure 6a is a C_60_ trimer that appeared on top of the first monolayer. It was found that all the three molecules in the second layer appeared at the atop position of the first C_60_ monolayer. Figure 6b shows a second layer island containing thirteen molecules arranged in a triangular pattern. Similar to the C_60_ trimer in Figure 6a, all thirteen C_60_ molecules also occupy the atop position of the first C_60_ layer, indicating the C_60_ molecules of second layer adopt the same hexagonal lattice as the first layer. Furthermore, we noticed that the intermolecular spacing for the peripheral molecules is slightly larger than that of the internal molecules. This is different from the second layer of C_60_ molecules on Au (111) that C_60_ molecules occupy the three-fold hollow sites of the first layer [46]. It is also different from the simple cubic lattice structure of C_60_ bulk below 249 K, or the face-centered cubic structure of C_60_ bulk above 249 K [51]. This phenomenon implies that the charge-transfer induced strong Coulomb interaction among fullerene molecules exist not only in the lateral directions, but also in the normal direction of the Cd substrate.

## 4. Conclusions

In summary, C_60_ molecules deposited on the Cd (0001) surface form a variety of domains with different misorientation angles with respect to the lattice directions of Cd (0001). Both orientation disorder and orientation ordering have been observed in the C_60_ domains. In the (2√3 × 2√3) R 30° domain, orientational disorder of the individual C_60_ molecules with either pentagon, hexagon, or 6:6 bond facing up has been observed. Orientation ordering appeared in the R26° domain such that all the C_60_ molecules adopt the same orientation with the 6:6 bond facing up. With the bias variation, possible orientation change of the C_60_ molecules takes place due to the influence from tip electrical field. In particular, complex chiral motifs composed seven C_60_ molecules with clockwise or anticlockwise handedness have been observed in the R4° and R8° domains, respectively. Due to the substrate screening and charge transfer from Cd to C_60_ molecules, the C_60_ molecules reveal a reduced HOMO–LOMO gap of 2.1 eV, which results in the strong Coulomb interaction among fullerene molecules in lateral and longitude directions. The orientational order and chiral superstructures found in present work provide essential information for understanding the C_60_-metal interaction, and are of great relevance to the carbon-based nanodevices and nanomaterials.
